# Chemical chaperones mitigate chronic short sleep induced cognitive impairment

**DOI:** 10.1002/alz.094751

**Published:** 2025-01-09

**Authors:** Kamalini Sengupta, Katya Scherbakova, Sydney Mason, Nirinjini Naidoo

**Affiliations:** ^1^ University of Pennsylvania, Philadelphia, PA USA

## Abstract

**Background:**

Epidemiological studies indicate that chronic short sleep and/or disrupted sleep are all associated with metabolic dysfunction, cardiovascular risk, cognitive impairments, and increased risk for Alzheimer’s disease. We have shown that acute sleep deprivation disrupts proteostasis, leading to the activation of an adaptive endoplasmic reticulum (ER) stress response known as the unfolded protein response (UPR). However, prolonged ER stress triggers the integrated stress response, which has been implicated in memory impairments. In this study, we investigated the effects of chronic short sleep (CSS) exposure in young adult wild‐type (WT) mice on learning and proteostasis in the presence and absence of chaperone therapy.

**Method:**

2‐month‐old male and female C57BL/6 mice underwent 4 weeks of CSS for 8hrs/day; 3 days/week by exposure to an enriched environment with or without 4‐phenyl butyrate (PBA) treatment. Control mice were left undisturbed, (n = 10/group/condition). Following CSS and recovery for 2 weeks mice were tested for hippocampal dependent learning. Brains were harvested for histology and biochemical analyses.

**Result:**

Spatial object recognition testing revealed no differences between any of the groups 2 weeks post CSS. However, SOR at 5 weeks post CSS revealed that the vehicle treated male CSS mice performed worse than the rested vehicle treated mice (p<0.02) and the PBA treated CSS mice performed better than the saline treated CSS mice (p<0.04). Similar results were observed in the female mice but at 7 weeks post CSS. Preliminary histological analyses for integrated stress markers indicate that PBA reduces PERK activation and signaling in the hippocampi of CSS mice. Analyses of other markers are in progress.

**Conclusion:**

Our data suggest that relieving proteostatic stress with chemical chaperones mitigates the effects of CSS on learning and memory.

